# Antidiabetic Activity and Chemical Composition of* Sanbai* Melon Seed Oil

**DOI:** 10.1155/2018/5434156

**Published:** 2018-05-02

**Authors:** Fang Wang, Haili Li, Hang Zhao, Ya Zhang, Pengcheng Qiu, Jie Li, Siwang Wang

**Affiliations:** ^1^Department of Natural Medicine, School of Pharmacy, Fourth Military Medical University, 169 West Changle Road, Xi'an, Shaanxi 710032, China; ^2^Xi'an Siyuan University, 28 Yong An Road, Xi'an, Shaanxi 710032, China; ^3^Yang Ling Fragrance Edible Oil Co., Ltd., Shaanxi 712100, China

## Abstract

**Objectives:**

Many fruits and herbs had been used in Traditional Chinese Medicines for treating diabetes mellitus (DM); however, scientific and accurate evidences regarding their efficacy and possible mechanisms were largely unknown.* Sanbai* melon seed oil (SMSO) was used in folk medicine in treating DM, but there is no literature about these effects. The present study was aimed at confirming the treatment effects of SMSO in type 1 DM.

**Methods:**

Diabetes was induced by single intraperitoneal injection of streptozotocin (STZ) at a dose of 65 mg/kg body weight. After diabetes induction, mice were treated with SMSO at dose of 1 g/kg, 2 g/kg, and 4 g/kg. Drugs were given by gavage administration once a day continuously for 28 days. At the end of treatment, several biochemical parameters and molecular mechanisms were determined by biochemical assays, ELISA, and Western blotting. The chemical compositions of SMSO were also tested.

**Results:**

SMSO treatment significantly improved the symptoms of weight loss, polydipsia, reduced FBG level, increased plasma insulin levels, reduced plasma lipids levels, and protected islet injury. The results also showed that SMSO mitigated oxidative stress and alleviated the liver and renal injury in diabetes mice. SMSO also protected islet cells from apoptotic damage by suppressing ER mediated and mitochondrial dependent apoptotic pathways. Further constituent analysis results showed that SMSO had rich natural resources which had beneficial effects on DM.

**Conclusions:**

This study showed that SMSO had excellent antidiabetes effect and provided scientific basis for the use of SMSO as the functional ingredients production and dietary supplements production in the food and pharmaceutical industries.

## 1. Introduction

Diabetes mellitus (DM) is always characterized by metabolic disorders of carbohydrates, fat, and protein. DM has been a severe threat to public health worldwide, not only in industrially developed countries, but also in the developing world like China [1]. In 2010, 285 million adults had DM, and the world prevalence was 6.4% among adults (20–79 years old). According to the survey, 439 million adults will have DM, about 7.7% in 2030 based on the theoretical calculations [2]. According to a cross-sectional survey with 98,658 Chinese adults in 2010, the prevalence of DM was 11.6%, which was a very high proportion [3]. DM leads to a series of chronic diabetic complications such as heart disease, kidney failure, retinopathy, neuropathy, and stroke [4]. One of the main causes for these complications was persistent hyperglycemia. In the diabetic patient, sustained hyperglycemia results in protein glycosylation and glucose autoxidation, which could cause excessive production of reactive oxygen species (ROS) in turn [5]. Some literatures also reported that the pancreatic beta-cell mass was reduced by oxidative stress in type 1 and type 2 diabetes [6, 7]. ROS could activate nuclear factor-*κ*B (NF-*κ*B) signaling pathway associated with inflammatory response. Inflammation plays a very important role in diabetes, insulin resistance, and complications [8, 9]. Therefore, the drugs which have antihyperglycemic and antioxidant activities may be useful for the treatment of DM.

In Traditional Chinese Medicine (TCM), DM is classified as “Xiao Ke” (consumption-thirst), and its classic symptoms include polydipsia, dry mouth, sweet urine, thirst, polyphagia, polyorexia, emaciation, and fatigue [10–13]. A number of TCM formulas or natural products were reported to have antidiabetic effects and some have been used in clinic for a long time [14–16]. In many African countries and other regions, plant medicine (phytomedicine) has been used in the management of diabetes [17]. Some vegetables and fruits were considered to be the main sources of various bioactive compounds which contain phenolic compounds, flavonoids, carotenoids, and ascorbic acid [18]. One of such fruits is* Citrullus lanatus*. It is a tropical fruit which is popular in the United States, Africa, Asia, and Russia.* Citrullus lanatus* is mainly harvested for juice and an excellent source of lycopene and vitamins [19]. However, the seeds are not eaten with juice and disposed as waste. In the seed of* Citrullus lanatus*, many phytochemical constituents were contained, like tannins, flavanoids, terpenoids, alkaloids, and others. Compared with other solvent flavanoids, flavanoids in seed had been reported to have many effects like antiinflammatory, antitumor, antioxidant, antiviral activities [20].

*Citrullus lanatus* cv. *Sanbai* (*Citrullus lanatus* (Thunb.) Matsum & Nakai cv.* Sanbai*), also known as* Sanbai* melon, is a kind of cultivated* Citrullus lanatus* species in China for a long history. The species is mainly distributed in Shanxi, Hebei, and Shaanxi Provinces. The ripe fruit is nearly globular with a white pulp, white peel, and white seeds. The seeds are bigger than that in other* Citrullus lanatus* and also have high oil content. In folk medicine, people like eating the fruit not only due to their delicious taste but also because they help to decrease blood glucose. There were several literatures which had showed that the* Citrullus lanatus* seeds had antioxidant, anti-inflammatory, and analgesic potential in DM animals [21–23]. In some Chinese ancient prescriptions like Compendium of Materia Medica (Bencao Gangmu), Chinese Materia Medica (Zhonghua Bencao), and Benjingfengyuan, it had been recorded that* Citrullus lanatus* seed had “Qingfei Runchang and Hezhong Zhike” effects, and it was also proved that* Citrullus lanatus* seed had antidiabetic activity. However, there is no literature about these effects of* Sanbai* melon seed oil (SMSO) and we do not know the chemical composition of SMSO. In this study, we want to determine the chemical composition of SMSO and illuminate its possible mechanism.

## 2. Materials and Methods

### 2.1. Materials

Squalene, *α*-tocopherol, *β*-tocopherol, *γ*-tocopherol, and *δ*-tocopherol were purchased from Sigma-Aldrich (St. Louis, MO, USA). Folin-Ciocalteu's phenol reagent was purchased from Merck (Darmstadt, Germany). High performance liquid chromatography- (HPLC-) grade methanol was purchased from Honeywell (Muskegon, MI, USA). Ultrapure water was prepared using a Millipore Milli-RO 12 plus system (Millipore Corp., Bedford, MA, USA). Streptozocin (STZ) was purchased from the Sigma-Aldrich (St. Louis, MO, USA). Antibodies were purchased from Santa Cruz Biotech. Inc., Santa Cruz, Calif., USA, and Cell Signaling Technology Inc., Danvers, MA. Other chemical reagents were all of analytical grade.

### 2.2. Plant Materials

Fresh fruits of* Sanbai* melon were obtained from Wanrong County, Yuncheng City, Shanxi Province, and botanically identified by Professor Wenzhe Liu, a botanist at the College of Life Sciences, Northwest University. Voucher specimen (FMMUDP-Voucher number SMS008) was deposited at the Department of Natural Medicine, School of Pharmacy, Fourth Military Medical University. The flesh and rinds were peeled off from the whole fruit and then seeds were collected, washed, and sun-dried. The seed oil extractions were kindly performed by Yangling Piaoxiang Edible Oil Co. Ltd.

### 2.3. Animals and Induction of Diabetes in the Experimental Animals

Male C57BL/6 mice weighing 20–23 g were obtained from the Experimental Animal Center of the Fourth Military Medical University. The experimental protocol was approved by the Ethics Committee for Animal Experimentation and was performed according to the Guidelines for Animal Experimentation of the Fourth Military Medical University and “the Guidance to Experimental Animal Welfare and Ethical Treatment” by The Ministry of Science and Technology of China. The animals were housed under a 12 h light-dark cycle and temperature was kept at 25°C.

The animals were acclimatized under laboratory conditions for one week prior to experiments. After overnight fasting, diabetes was induced by single intraperitoneal injection of streptozotocin (STZ) at a dose of 65 mg/kg body weight. STZ was freshly prepared and dissolved in 0.1 M sodium citrate buffer, pH 4.5. The fasting blood glucose level in the mice was determined by an Advanced Accu-Check Glucometer (Roche, Germany) after 3 days of injection. The mice with blood glucose ≥ 200 mg/dL were considered to be diabetic and were used for the further study.

### 2.4. Experimental Design

60 animals were randomly divided into five groups consisting of ten animals in each group: control group (CON); diabetic group (MOD); diabetic mice treated with 1 g/kg SMSO group (L); diabetic mice treated with 2 g/kg SMSO group (M); diabetic mice treated with 4 g/kg SMSO group (H); diabetic mice treated with 3 mg/kg glibenclamide (Gli). Drugs were given by gavage administration to their corresponding groups once a day continuously for 28 days. During the experimental period, blood glucose, body weight, and water intake of all mice were monitored weekly. On day 28, the mice were sacrificed following blood sample collection and the tissues of pancreas were dissected and stored at −20°C.

### 2.5. Determination of Fasting Blood Glucose Level and Insulin Level

The fasting blood glucose (FBG) level in the experimental mice was examined by an Advanced Accu-Check Glucometer (Roche, Germany) weekly during the treatment via tail prick method. The fasting serum insulin (FSI) level was measured by a radioactive immune analysis kit obtained from Beijing North Institute of Biological Technology (Beijing, China). The insulin content in pancreatic tissues was also measured according to the method used above.

### 2.6. Determination of Biochemical Parameters

Glycosylated hemoglobin (HbA1c) level in blood was determined according to the method reported with a dedicated ion exchange HPLC system (D-10™ Analyzer, Bio-Rad, USA) [26]. A 7180-automatic biochemical analyzer (Hitachi, Japan) was used to determine the serum levels of aspartate aminotransferase (AST), alanine transaminase (ALT), creatinine, urea, triglycerides (TG), total cholesterol (TC), and high density lipoprotein cholesterol (HDL-C) and low density lipoprotein cholesterol (LDL-C). The level of very low density lipoprotein cholesterol (VLDL-C) in serum was calculated by the Friedewald formula: VLDL = TG/5 [27].

### 2.7. Determination of Antioxidant Levels

Serum level of malondialdehyde (MDA) was assessed by a colorimetric reaction with thiobarbituric acid (TBA) according to the kit protocol. Activities of antioxidant enzymes superoxide dismutase (SOD), catalase (CAT), and reduced glutathione (GSH) levels were determined using commercial kits from Nanjing Jiancheng Bioengineering Institute (Nanjing, China).

### 2.8. Histopathological Examination

The excised pancreas tissues from the normal and experimental mice were immediately rinsed with normal saline and fixed in 10% buffered formalin. The tissues were cut into 5 *μ*m thick sections after embedding in paraffin and then stained with hematoxylin-eosin staining (HE) for histopathological examination.

### 2.9. Western Blot Analysis

Total proteins extracts of pancreas tissue were mixed with 2x SDS-PAGE sample buffer, boiled for 10 min. An equal amount of protein (30 *μ*g) from each sample was resolved by 10% SDS-PAGE being transferred onto polyvinylidene difluoride (Millipore, Billerica, MA, USA) membranes. After being blocked with 5% skim milk 37°C for 60 min, the blots were reacted with properly diluted monoclonal antibodies (1 : 1000) including PERK, P-PERK, elF2*α*, P-elF2*α*, cleaved caspase 12, cleaved caspase 3, Bcl-2, Bax, cleaved PARP, and *β*-actin. Following washing, the membranes were incubated with peroxidase-linked goat anti-rabbit IgG secondary antibody (1 : 1,000; Santa Cruz Biotechnology) for 1 h 37°C. Protein bands were detected using horseradish peroxidase-conjugated goat anti-mouse IgG antibodies followed by enhanced chemiluminescence reaction (Pierce Biotechnology, USA).

### 2.10. Chemical Composition Analysis of SMSO

The samples (1 mL) were diluted with HPLC-grade hexane (49 mL), and the solutions were filtered through a 0.22 *μ*m filter for gas chromatography mass spectrometry (GC-MS) analysis. The compositions of the samples were analyzed using an ISQ 110953 GC-MS system (ThermoFisher Corp, USA) equipped with a DB-5MS column (30 m × 0.25 mm × 0.25 *μ*m, Agilent Technologies). The analysis was operated under the following conditions: the temperatures of the injector, transfer line, and ion source were 300°C, 300°C, and 280°C, respectively. The amount of the sample injected was 1 *μ*L at a split ratio of 1 : 100. Helium (99.99%) was used as a carrier gas with a flow rate of 1 mL/min. The oven temperature was set at 60°C and held for 3 min at this temperature. Then, the temperature was raised to 300°C and held for 5 min; the flow rate was 3°C/min. Retention times were utilized as the primary criteria for the peak identifications. Compounds were identified using the MS spectral database (P/N: 274, 102, and 74 Thermo-Data System).

### 2.11. Quantification of Tocopherols

Tocopherols in the seed oils were quantified using the standard method of the agricultural industry of China (NY) (NY/T 1598-2008) with slight modifications. Tocopherols were analyzed by a Shimadzu (Kyoto, Japan) LC2010HT liquid chromatographic system comprising two LC-10ADvp pumps, an SIL-10ADvp autosampler, an SPD-10A(V)vp ultraviolet-visible detector, a CTO-10a(C)vp column oven, and an LC solution work station. HPLC analysis was performed on a Global Chromatography GS-120-5 C18 column (250 × 4.6 mm, 5 *μ*m, Unimicrotech). The mobile phase was methanol-ultrapure water, 98 : 2 (v/v), at a flow rate of 1 mL/min. The column temperature was maintained at 30°C. The detection wavelength was 294 nm. The volume of sample injected was 10 *μ*L. Quantification was performed based on calibration using standard tocopherols.

### 2.12. Determination of the Total Phenolic Content (TPC) and Total Flavonoid Content (TFC)

The extracts of samples were obtained following the method described before with slight modifications [28]. TPC in extracts were determined using the Folin-Ciocalteu assay with some modifications [29] and expressed as milligrams of gallic acid equivalents (GAEs) per 100 g oil (mg of GAEs/100 g oil). TFC in extracts was analyzed according to the procedures described by Ozsoy and coworkers with slight modifications [30]. The TFC was then expressed as mg of rutin equivalents (REs) per 100 g of oil (mg of REs/100 g oil).

### 2.13. The Quantification of Squalene

The amount of squalene was determined by an ISQ 110953 GC-MS system with a DB-5MS column. The chromatographic conditions were as follows: injector temperature 300°C, transfer line temperature 300°C, and ion source temperature 280°C. The amount of sample injected was 1 *μ*L at a split ratio of 1 : 10. Helium (99.99%) was used as a carrier gas with a flow rate of 1 mL/min. The oven temperature was set at 180°C and was then raised to 260°C with a flow rate of 15°C/min, raised to 280°C with a flow rate of 3°C/min, held for 2 min at 280°C, raised to 290°C with a flow rate of 10°C/min, and held for 3 min at 290°C. The retention time was utilized as the primary criterion for peak identification.

### 2.14. Determination of FAs

Fatty acids of SMSO were saponified and methyl-esterified according to ISO 5509:2000. The fatty acid methyl esters were identified by an ISQ 110953 GC-MS system with GSBP-INOWAX-MS column (30 m × 0.25 mm × 0.25 *μ*m, GS-TEK). Helium (99.999%) was used as the carrier gas at a constant flow rate of 1 mL/min. The injector temperature, transfer line temperature, and ion source temperature were 250°C, 280°C, and 250°C, respectively. The column was first set at 50°C, and the temperature was subsequently increased to 230°C at a rate of 5°C/min, where it was held for an additional 18 min. The amount of sample injected was 1 *μ*L. The 37 component FAMEs mixture was diluted 10-fold with n-hexane. The identification of the FAs in SMSO was conducted using a combination of retention time with the FAMEs mixture solution and the MS spectral database (P/N: 274, 102, and 74 Thermo-Data System).

### 2.15. Statistical Analysis

All data were expressed as mean ± SD. Statistical analysis was performed with SPSS 18.0 software (Armonk, NY, USA). Comparisons between groups were analyzed by one-way ANOVA, and group comparisons were analyzed using Student's independent sample *t*-test. Values of ≤0.05 were considered to be statistically significant.

## 3. Results

### 3.1. Effect of SMSO on Water Intake, Body Weight, FBG, and FSI Level

**Figure 1:**
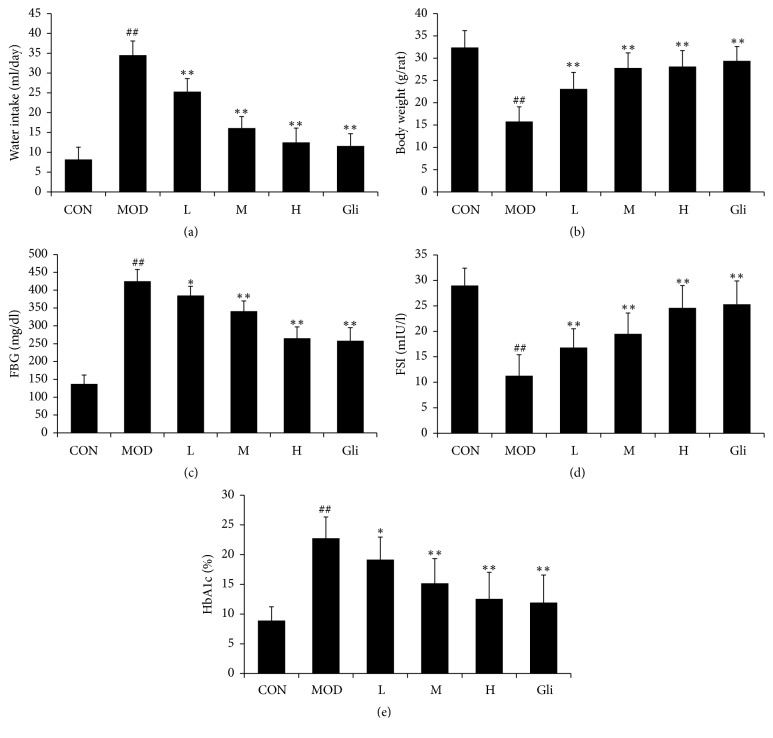
Effect of SMSO on water intake (a), body weight (b), FBG (c), and FSI (d) level and HbA1c ratio in diabetes mice. The values are expressed as mean ± SD (*n* = 10). ^##^*P* < 0.01 versus control group; ^*∗*^*P* < 0.05 and ^*∗∗*^*P* < 0.01 versus MOD group.

Body weight reduction, water intake increasing, hyperglycemia, and insulin level reduction were considered to be the typical characteristics of the diabetic pathophysiology. In this study, mice subjected to STZ showed all these changes; the results showed that the quantity of water intake was increased (Figure 1(a)), body weight was decreased to about 40% (Figure 1(b)), the FBG level was elevated to about 190% (Figure 1(c)), the FSI level was reduced to about 80% (Figure 1(d)), and HbA1c ratio was increased to about 23% (Figure 1(e)), suggesting that type 1 diabetes has been induced in these mice. However, treatment with SMSO improved the diabetic symptoms. SMSO treatment significantly enhanced the body weight, decreased the water intake, FBG level, and HbA1c ratio and increased FSI level in a dose dependent manner. These results suggested that SMSO had antidiabetic property in STZ induced diabetes model.

### 3.2. Effect of SMSO on Serum Lipid Profiles

The effect of SMSO on serum lipid levels in diabetes mice was showed in Table 1. As the results showed, the levels of TG, TC, LDL-C, and VLDL-C in MOD group were increased significantly by STZ in mice (*P* < 0.01), and HDL-C level was decreased slightly. However, treatment with SMSO on the diabetic mice caused significant decrease in TG, TC, LDL-C, and VLDL-C levels in diabetes mice. Compared with MOD group, HDL-C was also significantly increased in high dose of SMSO group and Gli group. These results suggested that SMSO inhibited the increased plasma lipids in STZ induced diabetes model.

### 3.3. Effect of SMSO on Pancreatic Damage

**Figure 2:**
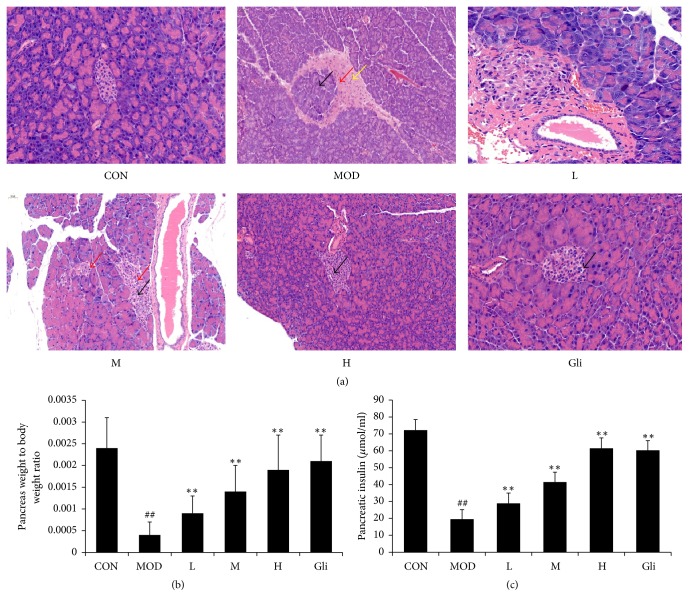
Effect of SMSO on pancreatic damage. (a) The pathological examination results of pancreatic islets in diabetic mice (HE 400x). (b) Effect of SMSO on pancreas weight to body weight ratio. (c) Effect of SMSO on insulin level in pancreas. The values are expressed as mean ± SD (*n* = 10). ^##^*P* < 0.01 versus control group;^*∗∗*^*P* < 0.01 versus MOD group. Islet atrophy is irregular and structurally disordered, as indicated by the black arrow; most of the islet cells had markedly decreased cytoplasm, as indicated by the red arrow; some of the islet cells had nuclear condensation and deep staining, as shown by the yellow arrow.

The results in Figure 2(a) showed histopathologic examination of pancreas in diabetes and normal mice. In CON group, appearance of islet cells was normal; however, in MOD group, number and size of pancreatic islets were decreased, and the pancreatic islets of Langerhans was disruption. Treatment with SMSO dose-dependently restored normal cellular appearance and size of the islet and enhanced islets regeneration. The weight of the pancreas in diabetes mice was also measured to evaluate the injury degree. As the results showed in Figure 2(b), pancreas weight to body weight ratio was significantly decreased in diabetes mice compared with that in normal mice, but SMSO treatment significantly increased the ratio and also elevated insulin level in pancreas (Figure 2(c)).

### 3.4. Effect of SMSO on Hepatic and Renal Function

ALT and AST were selected to evaluate the hepatic function, and urea and creatinine were selected to evaluate the renal function. As the results showed in Table 2, ALT, AST, urea, and creatinine levels in serum of diabetes mice were increased significantly when compared with the CON group. In SMSO treatment groups, ALT, AST, and creatinine were significantly decreased in a dose dependent manner. Low dose of SMSO caused a slight reduction of in urea level but had no significant difference. In middle and high dose group, urea level was decreased significantly compared with the MOD group. These results suggested that SMSO could improve hepatic and renal function in diabetes mice.

### 3.5. Effect of SMSO on Cellular Antioxidant Enzymes

**Figure 3:**
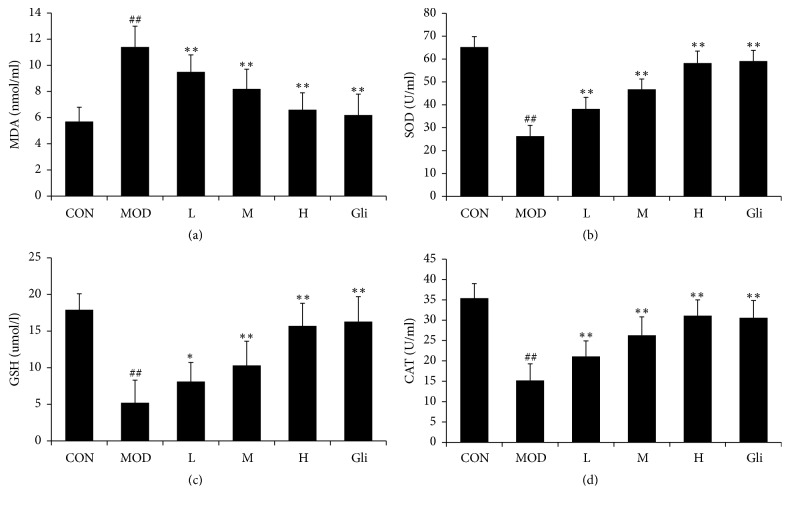
Effect of SMSO on cellular antioxidant enzymes and inflammatory cytokines. MDA (a), sod (b), CAT (c), and GSH (d) were measured in diabetes mice. The values are expressed as mean ± SD (*n* = 10). ^##^*P* < 0.01 versus control group; ^*∗*^*P* < 0.01 and ^*∗∗*^*P* < 0.01 versus MOD group.

In diabetes mice, MDA level was increased and activities of antioxidant enzymes (SOD, CAT, and GSH) levels were significantly decreased (Figures 3(a), 3(b), 3(c), and 3(d)). Mice treated with SMSO altered these changes in a dose dependent manner and brought those values near to normal. These results suggested that SMSO provided protection effects against STZ induced oxidative stress in diabetes mice.

### 3.6. Effect of SMSO on Apoptosis in Diabetes Mice

**Figure 4:**
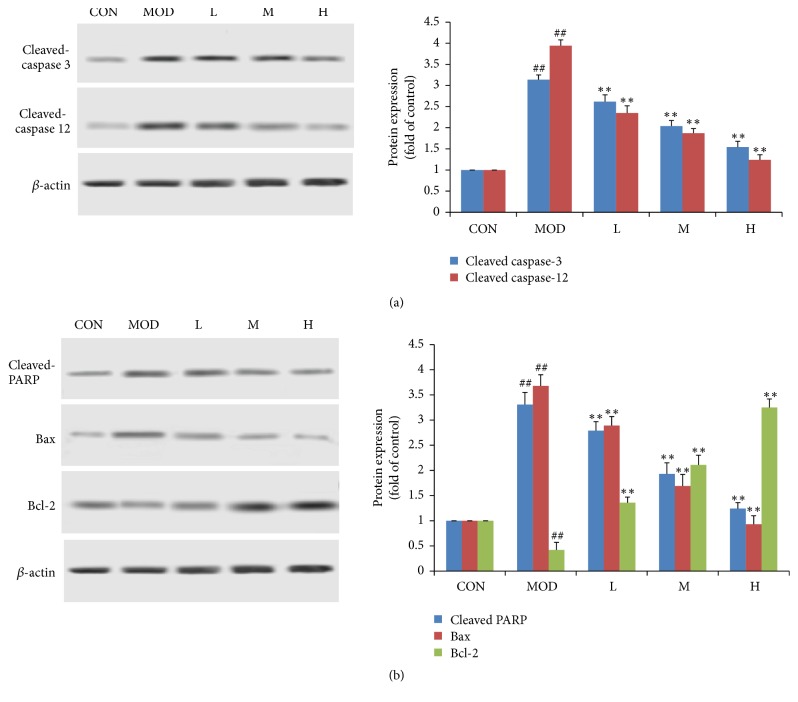
Effect of SMSO on ER related apoptosis and mitochondrial related apoptosis in diabetes mice. (a) Effect of SMSO on caspase 3 and caspase 12 cleavage in diabetes mice. (b) Effect of SMSO on Bcl-2, Bax, and cleaved PARP. The values are expressed as mean ± SD (*n* = 10).^##^*P* < 0.01 versus control group; ^*∗∗*^*P* < 0.01 versus MOD group.

Hyperglycemia induced endoplasmic reticulum (ER) stress and ER related apoptosis in diabetes mice. In this study, we found that caspase 12 and caspase 3 were cleaved and activated in MOD group, suggesting that apoptosis was induced through ER pathway (Figure 4(a)). In SMSO treatment group, caspase 3 and caspase 12 cleavage were significantly decreased in a dose dependent manner.

Next, mitochondrial dependent apoptotic pathway was tested to determine whether it was involved in STZ induced apoptosis. As the results in Figure 4(b) showed, Bax and cleaved PARP levels were increased, and Bcl-2 level was decreased in STZ treated mice, suggesting STZ induced apoptosis through mitochondrial dependent apoptotic pathway. In SMSO treatment group, Bax and cleaved PARP levels were decreased and Bcl-2 level was increased in a dose dependent manner.

### 3.7. Physicochemical Properties and Minerals of SMSO

The physicochemical properties of SMSO were showed in Table 3. As the results showed, the oil yield of SMSO was 26.14 ± 0.73%, which was similar to those reported for* Citrullus* (24.8 to 28.0%) [31] and higher than soybean seed oil (20.9%) [32]. In SMSO, unsaponifiable matter content was 5.87 ± 0.01 g/kg and saponification value was 197.31 ± 0.35 mg KOH/g oil which was higher than some vegetable oils, liking raspberry seed oil (191), grape seed oil (192.9) [33], olive oil (191.93), linseed oil (190.86), and sunflower oil (188.98) [34], suggesting SMSO might be useful in nonfood industries. The high iodine value reflects certain characteristics, including a high degree of unsaturation. In our study, the iodine value was 119.68 ± 1.20 g/100 g oil which was higher than the generally recommended amount for commercial vegetable oils, such as pumpkin seed oil (86.7 g/100 g) [35], soybean oil (74.8 g/100 g), and palm oil (53.2 g/100 g) (GB/T 5532-2008). The acid value and peroxide level were always used to control the quality of oil and value higher than 10 is harmful to the health. We found that the acid value of SMSO was 1.66 ± 0.10 mg KOH/g and peroxide level was 5.85 ± 0.07 mmol O_2_/kg oil, suggesting that SMSO was safe to eat. The minerals, including magnesium, iron, calcium, potassium, selenium, and chromium, were also detected and showed in Table 3.

### 3.8. The Results of Chemical Composition Analysis in SMSO

There have been no reports on the chemical composition of SMSO. In this study, the chemical compositions of SMSO were analyzed by GC-MS with the data available using the MS spectral database (P/N: 274, 102, and 74 Thermo-Data System). The results showed that SMSO had a very complex composition (the identified compounds are reported in Table 4). We found that SMSO contained various FAs, squalene, tocopherols, TPC, and TFC, and these compositions were analyzed in the further study.

### 3.9. FAs Contents in SMSO

Sixteen FAs were detected in SMSO and showed in Table 5. As the results showed, the principal FAs were linoleic acid (52.82 ± 2.68%), followed by oleic acid (24.13 ± 2.22%) and palmitic acid (13.69 ± 2.07%), and these three FAs represent more than 98.6% of the total FAs of SMSO. The percentages of saturated fatty acid (SFA), monounsaturated fatty acid (MUFA), and polyunsaturated fatty acid (PUFA) were similar to those of commonly consumed vegetable oils, such as soybean, corn, and sunflower. High levels of unsaturated fatty acid (UFA) were found in the sample, and the levels of SFA were low in the sample. Linoleic acid, as *ω*-6 PUFA, decreases the levels of total plasma cholesterol, has favorable effects on the prevention of coronary heart diseases and cancers, and increases insulin sensitivity. In this study, we found that the concentration of linoleic acid was the highest (52.82 ± 2.68%) besides the FAs and higher than that of soybean (50.17 ± 0.83%) and corn (49.27 ± 1.24%).

### 3.10. The Quantification of Squalene, Tocopherols, TPC, and TFC in SMSO

Squalene, tocopherols, TPC, and TFC are widely recognized as natural antioxidant substances, and we measured these compounds in SMSO (Table 6). The amount of squalene was determined via a GC-MS system with a DB-5MS column and calculated using a calibration curve of squalene standard solutions (5.86–586 mg/L) with *y* = 677153.9006*x* − 2380914.6696  (*R*^2^ = 0.9998). As the results showed in Table 6, the amount of squalene was 753.02 ± 27.13 mg/kg in SMSO. The amount of total tocopherols was 65.78 ± 0.49 mg/100 g, which contained *α*-tocopherol (6.81 ± 1.26 mg/100 g), (*β* + *γ*)-tocopherol (57.52 ± 0.37 mg/100 g), and *δ*-tocopherol (1.44 ± 0.36 mg/100 g). The TPC and TFC contents of were 638.53 ± 10.12 mg/100 g and 322.93 ± 12.80 mg/100 g.

## 4. Discussion

All over the world, diabetes mellitus (DM), one of the most common metabolic disorders, has been considered as one of the most serious diseases threatening human health. DM is characterized by the destruction or dysfunction of islet cells which caused no enough insulin or no response to the insulin [36]. Hyperglycemia is considered as one of the main features of DM and causes several diabetes complications in the body [37]. Sustained hyperglycemia induces oxidative and ER stresses which further enhance important organs damage. Some reports also found that oxidative and ER stress could cause the pancreas islet function impairment and lead to hyperglycemia [38]. In addition, some researchers found that DM was associated with inflammatory response [39]. Thus, it is necessary to develop an ideal antidiabetic drug with reducing blood glucose as well as antioxidant and anti-inflammatory properties. Until now, several medicines were taken to treat DM, but the outcome is not that satisfactory. So it is time to develop some new alternative approaches. Traditional Chinese Medicine (TCM) has been selected and used in treating DM for thousands of years, characterized by low cost, accessibility, simplicity, and efficacy [40]. In many cases, TCM was used as empirical medicine and had little scientific evidence to verify how TCMs work under the disease condition [41]. It is necessary to systematically evaluate the efficacy of TCMs.

Some fruits have been used as medicine in TCM, such as* Citrullus lanatus*, a popular fruit in many countries. Previous researches had found that* Citrullus lanatus *had a beneficial effect on diabetes [42]. According to the results of constituent analysis, the seed of* Citrullus lanatus *contains abundant phytochemical constituents and flavanoids, which have many effects like anti-inflammatory, antitumor, antioxidant, and antiviral activities.* Sanbai* melon (*Citrullus lanatus* (Thunb.) Matsum & Nakai cv.* Sanbai*) is a kind of cultivated* Citrullus lanatus* species, and in folk medicine local people use it as fruit and antidiabetic.* Sanbai* melon seed oil (SMSO) is used as edible oil in cooking and also has hypoglycemic activity. However, there was little research on the efficacy of SMSO as an antidiabetic agent and the beneficial role in diabetic complications. In this study, we tried to determine protective effects of SMSO on DM and its chemical composition.

Body weight reduction, water intake increasing, hyperglycemia, and insulin level reduction have been considered as the typical characteristics of diabetes. Our results were consistent with these characteristics. In rats treated with STZ, beta-cells were destructed by excess ROS, and insulin production was reduced, in turn enhancing blood glucose level. Excess ROS also caused excessive breakdown of the muscle tissues, fats, and proteins, thus inducing body weight loss [43]. Treatment with SMSO altered these changes, suggesting SMSO had antidiabetic activity. HbA1c was always used as a reliable index for blood glucose control in diabetes. High level of HbA1c indicated high risks for the development and/or progression of diabetic complications [44]. In this study, high level of HbA1c was observed in diabetes rats, and administration of SMSO significantly reduced HbA1c levels. We also found lipid metabolism disorders in diabetic rats. Previous study showed that regulation of lipid metabolism could decrease the risk of microvascular disease and related complications [45]. SMSO significantly decreased the TG, TC, LDL-C, and VLDL-C levels in a dose dependent manner, suggesting SMSO could prevent the hyperlipidemia associated with diabetes. These results indicated that SMSO could regulate glucose and lipid metabolism disorder in diabetes rats.

Histopathological examination indicated that STZ treatment destroyed pancreatic islets and thus resulted in pancreatic beta-cells insulin secretion and beta-cell mass reduction significantly and led to diabetes (type 1 reduced 70% nearly and type 2 reduced 50% nearly) [46]. In this study, we observed that amount of pancreatic beta-cells, weight of the pancreas, and insulin level in pancreas were reduced in diabetes mice. However, SMSO treatment effectively restored these adverse effects in a dose dependent manner. Hepatic and renal function was all very important to diabetic and normal people. ALT and AST are two important markers in liver function evaluation. Elevated levels of these two indicators in serum indicated that the liver function was injured [47].

Kidney plays important role in removing the metabolic waste from body and maintaining body homeostasis [48]. Urea and creatinine are two important markers in renal function evaluation. Increased levels of ALT, AST, urea, and creatinine were observed in diabetes mice, suggesting impaired hepatic and renal dysfunction. Treatment of diabetic mice with SMSO and glibenclamide significantly reduced levels of ALT, AST, creatinine, and urea, suggesting SMSO could alleviate the liver and renal injury in diabetes mice.

In DM, excessive glucose and free fatty acids (FFA) levels could induce oxidative stress and further induce diabetes or make it become more serious through various mechanisms [48, 49]. In addition, excessive oxidative stress also induces or promotes the diabetes related chronic diseases, such as diabetic cardiomyopathy, diabetic nephropathy, and diabetic neuropathy [50]. As the literature showed, oxidative stress has close relationship with inflammation, and they closely relate to each other [51, 52]. When oxidative stress was induced in DM, it could stimulate various proinflammatory cytokines' production in the adipose tissue [53]. In our study, oxidative stress was induced by STZ treatment, according to the results of antioxidant enzymes. After treatment with SMSO, the levels of MDA were significantly decreased and the levels of SOD, CAT, and GSH were increased in a dose dependent manner. These results strongly supported that SMSO had antioxidant effects, and hence SMSO might be used in reducing or preventing diabetes related micro- and macrovascular complications.

Literature suggested that oxidative stress, inflammation, hyperglycemia, ER stress, and apoptosis are interrelated closely [54]. Apoptosis includes mitochondrial dependent apoptotic pathway and ER related apoptosis. At the normal condition, ER in pancreatic beta-cells plays some important roles in producing, folding, and exporting newly synthesized insulin [55]. When cells were subjected to hyperglycemia, high lipid load, and oxidative stress, ER homeostasis is disturbed and induced ER stress. Serious and longtime ER stress caused ER unable to keep normal cellular function and then triggered cell apoptosis or death [56]. In this study, we found that cleaved caspase 12 (an ER stress associated protein) and cleaved caspase 3 were upregulated in the STZ treatment group. In the SMSO treatment group, cleaved caspase 12 and cleaved caspase 3 were successfully reduced, showing its response against ER stress. Mitochondrial dependent apoptotic pathway was characterized by mitochondrial membrane potential reduction, cytochrome-C leakage from mitochondria, apoptosome formation, increasing of caspase 9, caspase 3, and PARP cleavage, and Bax/Bcl-2 ratio imbalance [57]. In this study, Bax, Bcl-2, and cleaved PARP levels were measured and it was found that Bax and cleaved PARP levels, which were increased by STZ, were decreased and Bcl-2 levels were increased, showing its response against mitochondrial dependent apoptosis.

To analyze the components in SMSO and its physicochemical properties, various oils' properties, minerals, FAs, squalene, tocopherols, TPC, and TFC were measured. The results showed that SMSO possessed similar physicochemical properties, constituent minerals, major chemical compounds, and FAs, which might be useful in food and nonfood applications. In particular, the FAs and the percentages of SFA, MUFA, and PUFA were similar to those of some commonly consumed vegetable oils. These abundant nutritional materials associated with the desirable sensory characteristics of SMSO provided incentive for the industrial production and commercialization of* Sanbai* melon seed. Squalene, tocopherols, TPC, and TFC are widely recognized as natural antioxidant substances and found in the SMSO. Squalene is a triterpene belonging to the terpenoid family and has antioxidative, hypotriglyceridemic, and hypoglycemic effect [58]. Tocopherol which has excellent antioxidant capacity plays an important role as an antioxidant in many diseases, including diabetes and cardiovascular diseases [59]. Total phenolics and flavonoids are the most used natural antioxidant substances in TCM and show beneficial effects on the development of diabetes and cardiovascular diseases [60]. Squalene, tocopherols, TPC, and TFC were abundant in the SMSO, suggesting that the antidiabetic effects of SMSO might be induced by these compounds. The results showed that SMSO had rich natural resources, suggesting that SMSO might be used for the functional ingredients production and dietary supplements production in the food and pharmaceutical industries.

In conclusion, the results of this study confirmed that SMSO had excellent antidiabetic effect, characterized by its antihyperglycemic and hypolipidemic effects, attributed to its antioxidative stress, and protected islet cells from apoptotic damage by suppressing ER mediated and mitochondrial dependent apoptotic pathways. Further constituent analysis results showed that SMSO had rich natural resources which had beneficial effects on DM. All these findings from this study provide scientific basis for the use of SMSO in the functional ingredients production and dietary supplements production in the food and pharmaceutical industries.

## Figures and Tables

**Table 1:** Effect of CSSO on serum lipid profiles.

Groups	TC (mmol/L)	TG (mmol/L)	HDL-C (mmol/L)	LDL-C (mmol/L)	VLDL-C (mmol/L)
CON	0.91 ± 0.12	0.41 ± 0.15	0.64 ± 0.13	0.19 ± 0.07	0.13 ± 0.05
MOD	2.98 ± 0.23^##^	2.34 ± 0.24^##^	0.31 ± 0.24^##^	0.63 ± 0.12^##^	0.41 ± 0.08^##^
L	2.14 ± 0.19^*∗*^	1.91 ± 0.33^*∗∗*^	0.36 ± 0.21	0.53 ± 0.11^*∗*^	0.32 ± 0.07^*∗*^
M	1.57 ± 0.31^*∗∗*^	1.25 ± 0.39^*∗∗*^	0.41 ± 0.24^*∗*^	0.41 ± 0.09^*∗∗*^	0.24 ± 0.06^*∗∗*^
H	1.24 ± 0.28^*∗∗*^	0.87 ± 0.27^*∗∗*^	0.66 ± 0.19^*∗∗*^	0.27 ± 0.12^*∗∗*^	0.19 ± 0.07^*∗∗*^
Gli	1.21 ± 0.33^*∗∗*^	0.91 ± 0.31^*∗∗*^	0.63 ± 0.24^*∗∗*^	0.24 ± 0.11^*∗∗*^	0.16 ± 0.05^*∗∗*^

^##^ *P* < 0.01 versus CON group; ^*∗*^*P* < 0.05 and ^*∗∗*^*P* < 0.01 versus MOD group.

**Table 2:** Effect of SMSO on hepatic and renal function.

Groups	ALT (U/L)	AST (U/L)	Urea (mmol/L)	Creatinine (*μ*mol/L)
CON	15.63 ± 4.16	42.94 ± 7.18	5.66 ± 1.13	45.71 ± 5.88
MOD	63.38 ± 5.72^##^	114.77 ± 8.32^##^	11.72 ± 1.21^##^	77.49 ± 6.11^##^
L	51.17 ± 6.25^*∗∗*^	92.55 ± 7.99^*∗∗*^	10.57 ± 1.42	63.18 ± 5.11^*∗∗*^
M	33.72 ± 6.19^*∗∗*^	72.17 ± 9.14^*∗∗*^	8.25 ± 0.97^*∗∗*^	55.67 ± 4.97^*∗∗*^
H	23.61 ± 5.94^*∗∗*^	56.17 ± 8.82^*∗∗*^	6.55 ± 1.31^*∗∗*^	48.92 ± 5.36^*∗∗*^
Gli	22.19 ± 6.02^*∗∗*^	55.62 ± 8.36^*∗∗*^	6.79 ± 1.22^*∗∗*^	47.19 ± 4.68^*∗∗*^

^##^ *P* < 0.01 versus CON group; ^*∗*^*P* < 0.05 and ^*∗∗*^*P* < 0.01 versus MOD group.

**Table 3:** Physicochemical properties and minerals in SMSO.

Items	Unit	SMSO
Oil yield	%	26.14 ± 0.73
Refractive index (20°C)		1.473 ± 0.004
Relative density		0.928 ± 0.001
Unsaponifiable matter	g/kg	5.87 ± 0.01
Saponification value	KOH/g	197.31 ± 0.35
Iodine value	g/100 g	119.68 ± 1.20
Peroxide value	mmol O_2_/kg	5.85 ± 0.07
Acid value	mg KOH/g	1.66 ± 0.10
Selenium	*µ*g/100 g	8 ± 1.00
Potassium	mg/100 g	0.98 ± 0.16
Calcium	mg/100 g	0.82 ± 0.15
Iron	mg/100 g	1.4 ± 0.50
Magnesium	mg/100 g	0.89 ± 0.10
Chromium	mg/100 g	0.091 ± 0.01
Manganese	mg/100 g	nd
Mercury	mg/100 g	nd
Lead	mg/100 g	0.009 ± 0.002
Arsenic	mg/100 g	0.008 ± 0.003

nd: not detected.

**Table 4:** Chemical composition of SMSO.

Peak number	Retention time (min)	Molecular formula	Name of chemical compound	SI	Area%
(1)	7.61	C_7_H_12_O	(E)-2-Heptenal	770	0.18
(2)	22.11	C_10_H_16_O	(E,E)-2,4-Decadienal	781	0.32
(3)	28.01	C_14_H_28_O_2_	Tetradecanoic acid	849	3.61
(4)	32.86	C_16_H_22_O_4_	1,2-Benzenedicarboxylic acid, bis(2-methylpropyl) ester	925	3.26
(5)	39.75	C_15_H_22_O_3_	2-Ethylhexyl salicylate	904	2.11
(6)	40.86	C_17_H_34_O_2_	Isopropyl myristate	923	1.51
(7)	42.57	C_15_H_30_O_2_	Pentadecanoic acid	894	1.45
(8)	44.32	C_18_H_34_O_2_	Oleic acid	800	0.7
(9)	45.49	C_16_H_30_O_2_	Palmitoleic acid	890	1.83
(10)	45.69	C_16_H_22_O_4_	Dibutyl phthalate	889	0.91
(11)	46.75	C_16_H_32_O_2_	n-Hexadecanoic acid	925	7.1
(12)	50.07	C_12_H_22_O_2_	Heptanoic acid, 2-penten ester	888	3.62
(13)	50.43	C_19_H_38_	1-Nonadecene	906	1.01
(14)	52.20	C_18_H_32_O_2_	(Z,Z)-9,12-Octadecadienoic acid	841	10.56
(15)	52.45	C_18_H_34_O_2_	(E)-9-Octadecenoic acid	863	3.85
(16)	53.23	C_18_H_36_O_2_	Octadecanoic acid	830	1.76
(17)	53.48	C_16_H_33_NO	Hexadecanamide	798	1.06
(18)	55.92	C_24_H_38_O_4_	Diisooctyl phthalate	890	1.52
(19)	57.26	C_20_H_32_O_2_	Benzoic acid, tridecyl ester	846	3
(20)	57.53	C_18_H_26_O_3_	2-Propenoic acid, 3-(4-methoxyphenyl)-2-ethylhexyl ester	951	3.54
(21)	58.49	C_19_H_34_O_2_	Methyl 5,12-octadecadienoate	846	4.11
(22)	58.69	C_18_H_35_NO	(Z)-9-Octadecenamide	831	0.72
(23)	59.46	C_18_H_37_NO	Octadecanamide	877	0.84
(24)	60.20	C_21_H_34_O_2_	Benzoic acid, tetradecyl ester	880	0.87
(25)	61.75	C_21_H_36_O_4_	(Z,Z,Z)-9,12,15-Octadecatrienoic acid, 2,3-dihydroxypropyl ester	784	2.05
(26)	61.99	C_25_H_40_O_6_	(Z,Z,Z)-9,12,15-Octadecatrienoicacid,2-(acetyloxy)-1-[(acetyloxy)methyl] ethyl ester	785	1.6
(27)	62.71	C_16_H_34_S	tert-Hexadecanethiol	770	0.77
(28)	62.92	C_19_H_38_O_4_	Hexadecanoic acid, 2-hydroxy-1-(hydroxymethyl) ethyl ester	825	0.62
(29)	63.05	C_22_H_36_O_2_	Benzoic acid, pentadecyl ester	791	1.21
(30)	63.40	C_24_H_38_O_4_	Diisooctyl phthalate	923	1.91
(31)	66.55	C_37_H_76_O	1-Heptatriacotanol	805	0.6
(32)	67.43	C_21_H_38_O_4_	(Z,Z)-9,12-Octadecadienoic acid, 2-hydroxy-1-(hydroxymethyl) ethyl ester	833	0.95
(33)	67.54	C_21_H_40_O_4_	(Z)-9-Octadecenoic acid, 2-hydroxy-1-(hydroxymethyl) ethyl ester	829	0.96
(34)	70.5	C_30_H_50_	Squalene	946	15.47
(35)	76.67	C_27_H_46_O	Cholesterol	905	2.17
(36)	80.68	C_29_H_50_O_2_	Tocopherol	844	1.68
(37)	81.18	C_34_H_66_O_2_	(Z)-9-Hexadecenoic acid, octadecyl ester	831	1.91

**Table 5:** Fatty acid compositions (FAs) (% of total fatty acids) in SMSO.

FAs	FA content (%)			
	SMSO	Soybean	Corn [24]	Sunflower [25]
*Saturated*				
C14:0	0.12 ± 0.01	0.12 ± 0.03	0.05 ± 0.01	0.08 ± 0.02
C16:0	13.69 ± 2.07	15.65 ± 0.03	13.34 ± 0.50	6.44 ± 0.37
C17:0	0.12 ± 0.02	0.14 ± 0.03	0.09 ± 0.02	0.07 ± 0.04
C18:0	5.29 ± 0.22	4.98 ± 0.23	1.91 ± 0.10	5.02 ± 0.84
C20:0	0.56 ± 0.09	0.55 ± 0.07	0.46 ± 0.04	0.33 ± 0.05
C22:0	0.15 ± 0.03	0.34 ± 0.04	0.23 ± 0.10	0.83 ± 0.08
C23:0	0.39 ± 0.06	nd	nd	nd
C24:0	0.15 ± 0.05	nd	0.22 ± 0.07	0.28 ± 0.04
*Monounsaturated*				
C16:1n7	0.15 ± 0.02	0.12 ± 0.03	0.11 ± 0.02	0.09 ± 0.02
C17:1n7	0.03 ± 0.01	0.09 ± 0.02	0.07 ± 0.04	0.03 ± 0.01
C18:1n9	24.13 ± 2.22	20.98 ± 0.23	31.77 ± 0.85	25.29 ± 1.83
C20:1n9	0.23 ± 0.07	0.32 ± 0.06	0.26 ± 0.03	0.15 ± 0.02
C22:1n9	0.19 ± 0.04	0.38 ± 0.08	nd	0.04 ± 0.06
*Polyunsaturated*				
C18:2n6	52.82 ± 2.68	50.17 ± 0.83	49.27 ± 1.24	60.03 ± 2.23
C18:3n3	0.51 ± 0.16	8.18 ± 0.53	0.65 ± 0.10	0.14 ± 0.07
C22:2n6	0.06 ± 0.02	nd	nd	nd
∑SFA	20.48 ± 2.24	21.89	16.30 ± 0.50	13.05 ± 0.92
∑MUFA	24.73 ± 2.18	22.11	32.98 ± 2.88	26.27 ± 1.95
∑PUFA	53.39 ± 2.61	58.86	50.72 ± 1.19	60.68 ± 2.08

nd: not detected.

**Table 6:** Tocopherols, total phenolic content (TPC), and total flavonoid content (TFC) in SMSO.

Squalene (mg/kg)	Tocopherols (mg/100 g)				TPC (mg GAEs/100 g)	TFC (mg REs/100 g)
	*α*-Tocopherol	(*β* + *γ*)-Tocopherol	*δ*-Tocopherol	Total		
753.02 ± 27.13	6.81 ± 1.26	57.52 ± 0.37	1.44 ± 0.36	65.78 ± 0.49	638.53 ± 10.12	322.93 ± 12.80
